# State-level income inequality and the odds for meeting fruit and vegetable recommendations among US adults

**DOI:** 10.1371/journal.pone.0238577

**Published:** 2020-09-09

**Authors:** Masako Horino, Sze Yan Liu, Eun-Young Lee, Ichiro Kawachi, Roman Pabayo

**Affiliations:** 1 Johns Hopkins Bloomberg School of Public Health, Baltimore, MD, United States of America; 2 School of Community Health Sciences, University of Nevada, Reno, Reno, NV, United States of America; 3 Public Health Department, Montclair State University, New York, NY, United States of America; 4 Weill Cornell Medical College, New York City, NY, United States of America; 5 School of Kinesiology & Health and Department of Gender Studies, Queen's University Kingston, ON, Canada; 6 Department of Social Behavioral Sciences, Harvard T.H. Chan School of Public Health, Boston, MA, United States of America; 7 School of Public Health, University of Alberta, Edmonton, AB, Canada; University of British Columbia, CANADA

## Abstract

**Background:**

Previous research indicates that income inequality is associated with risk for mortality, self-rated health status, chronic conditions, and health behavior, such as physical activity. However, little is known about the relationship between income inequality and dietary intake, which is a major risk factor for common chronic diseases including heart disease, stroke, diabetes, and certain types of cancers. The objective of this study is to determine the association between US state income inequality and fruit and vegetable consumption among adults.

**Methods:**

Cross-sectional data on 270,612 U.S. adults from the U.S. 2013 Behavioral Risk Factor Surveillance System was used. Fruit and vegetable consumption was assessed from the six-item fruit and vegetable frequency questionnaire, which is part of the Behavioral Risk Factor Surveillance System. Multilevel modeling was used to determine whether US state-level income inequality (measured by the z-transformation of the Gini coefficient) was associated with fruit and vegetable consumption adjusting for individual-level and state-level covariates.

**Results:**

In comparison to men, women were more likely to consume fruits and vegetables ≥5 times daily, fruits ≥2 times daily, vegetables ≥3 times of daily, and less likely to consume fruit juice daily. Among both men and women, a standard deviation increase in Gini coefficient was associated with an increase in consuming fruit juice daily (OR = 1.07, 95% CI = 1.03, 1.11). However, among women, a standard deviation increase in Gini coefficient was associated with a decreased likelihood in meeting daily recommended levels of both fruits and vegetables (OR = 0.93; 0.87–0.99), fruits only (OR = 0.95; 95% CI, 0.92–0.99) and vegetables only (OR = 0.92; 95% CI, 0.89–0.96).

**Conclusions:**

This study is one of the first to show the relationship between income inequality and fruit and vegetable consumption among U.S. adults empirically. Women’s health is more likely to be detrimentally affected when living in a state with higher income inequality.

## Introduction

In the United States, approximately half of adults suffer from one or more chronic diseases [1]. An adequate amount of fruit and vegetable consumption is associated with reduced risk of common chronic diseases including heart disease, stroke, diabetes, and certain types of cancers [2–5]. Over the last few decades, given the known benefits of adequate fruit and vegetable consumption, a major theme of U.S. dietary guidelines focused on increasing consumption of nutrient-rich foods, with an emphasis on dark green, orange, and red vegetable subgroups, along with peas and beans [6]. Despite its known benefits and continuous promotion on consumption of fruits and vegetables, very few Americans meet the recommended level of fruit and vegetable intake and there is a considerable variation on fruit and vegetable intake across the U.S. states [7]. There is debate about whether fruit juice should count towards fruit intake recommendations. While 100% fruit juice can be counted toward fruit intake [6], moderate consumption is recommended due to its low fiber and high sugar content, and its association with an increased risk of type 2 diabetes [8] and greater weight gain over time [9]. The proportion of state populations meeting recommendations varied from 7.0% (West Virginia) to 18.1% (District of Columbia) for fruits and from 4.7% (Louisiana) to 11.5% (Oregon) for vegetables [7].

Previous literature has identified three broad levels playing a critical role in fruit and vegetable consumption among the U.S. population; individual, local community and macro-levels [10]. According to the socio-ecological model, macro-level factors may have the most profound impact on fruit and vegetable consumption at the population level. The macro-level determinants of nutrition include social, historical, and political factors [11], and influence access to and availability of fruits and vegetables through food system and policy, agriculture, manufacture, distribution and cost. These factors also influence distribution of wealth and income within societies, private resources available to individuals [12], affecting distribution of poverty and food insecurity, ultimately, influencing population’s health, disease trends, and health behavior and dietary quality. Numerous previous studies have reported increasing disparities in fruit and vegetable intake across the socioeconomic strata in the U.S. [13], Korea [14], and Denmark [15]; however, limited evidence exists on the individual-level relationship between distribution of wealth and income and dietary behavior at societal level.

Income inequality has been identified as a risk factor of numerous health outcomes, such as mortality and self-rated health status [16–18]. Recent studies also have provided substantial evidence on the relationships between income inequality, chronic conditions, and health behavior [19, 20]. Specifically, a detrimental association between income inequality and health outcome was found in several ecological studies and a few longitudinal studies using large-area aggregate measures, such as country- or state-level income inequality. Specifically, among the developed countries, income inequality was significantly related to the prevalence of obesity both among men and women, diabetes mortality, per capital calorie consumption and mean Body Mass Index (BMI) [21, 22]. In the U.S., higher income inequality at the state level has been linked with increased prevalence of overweight/obesity, hypertension, sedentary behavior, and heart attack [19, 20, 23].

It has been widely accepted that distribution of wealth and income within the society affects population health by independently operating from the absolute position of an individual in social ladder [24]. Previous studies have found that societies with large social inequalities have significantly poorer health outcomes when compared to those with more egalitarian societies, even after controlling for area-level variables [23, 24]. Given these, a possible mechanism between income inequality and chronic diseases could potentially be the effect of health behaviors. We previously identified an association between income inequality with physical activity behavior among women in the U.S. [19]. Income inequality may also be associated with dietary intake, such as fruit and vegetable intake. In other words, income inequality might influence risk for obesity, heart attack and other chronic morbidities through dietary intake. A first step would be to identify an association between income inequality and dietary behavior, such as fruit and vegetable intake. In a recent study, researchers did not observe a significant relationship between income inequality and fruit and vegetable intake but these findings were observed among adults in Colombia [25]. Income inequality might be adversely associated with fruit and vegetable intake among adults living in higher socioeconomic countries.

There is growing evidence that income inequality at U.S. state-level is associated with chronic conditions. However, what has not been studied extensively is the association between the level of income inequality and health behaviors such as fruit and vegetable intake. Given that dietary intake is one of the risk factors for many chronic diseases, research is needed to determine whether income inequality affects fruit and vegetable consumption of individuals. If the income inequality is associated with lower fruit and vegetable consumption, then further understanding of the contextual pathways in which income inequality affects fruit and vegetable consumption would inform effective strategies and policies to improve Americans’ dietary quality. The purpose of this study is to examine the associations between state-level income inequality and frequency of fruit and vegetable consumption in the U.S. We used a cross-sectional study design and a population-based sample to test the hypotheses that income inequality at state level is inversely associated with fruit and vegetable consumption.

## Methods

### Sample

The data came from 2013 Behavioral Risk Factor Surveillance System, which is a random-digit dialed telephone survey conducted by the Center for Disease Control and Prevention. The Behavioral Risk Factor Surveillance System collects health risk data from all 50 states, the District of Columbia, Guam, Puerto Rico, and the Virgin Islands [26]. For this investigation, data from respondents residing in the 50 states and the District of Columbia were used. The target population of Behavioral Risk Factor Surveillance System includes non-institutionalized individuals aged ≥18 years with access to a landline or a cellular telephone. The University of Nevada, Reno Institutional Review Board reviewed this project and has determined it is EXEMPT from IRB review according to federal regulations and University policy.

### Patient and public involvement

Secondary data were utilized for this project. Therefore, we did not involve patients or the public in the design of this study.

### Measures

#### Area-level covariates

The main exposure of interest is state-level income inequality, measured using the Gini coefficient, which was calculated using 2010 U.S. Census data in each of the 50 states and the District of Columbia [27]. Mathematically, the Gini coefficient is defined as half of the arithmetic average of the absolute differences between all pairs of incomes in a population [17]. The Gini coefficient is a measure that represents one-half of the average difference in income between any two individuals randomly sampled from the distribution, in which being normalized on mean income [17]. The Gini coefficient theoretically ranges from 0 (perfect equality, where every household earns exactly the same income) to 1.0 (perfect inequality) [17]. Detailed calculation of the Gini coefficient has been described previously [17]. Although various measures of relative inequality within a given residential area exist, the Gini coefficient is the most frequently used in public health research [28–30]. Other commonly used measures of income inequality include the 90:10 income ratio, Theil entropy index, and the Atkinson index. However, at the state level, these have been shown to be correlated with the Gini above 0.9 [16].

Other state-level covariates include median income, proportion of the state in poverty, proportion of the state population that is African American, population size, and census regional divisions of the United States (New England as a reference category, Middle Atlantic, East North Central, West North Central, South Atlantic, East North Central, West South Central, Mountain, and Pacific). Z-transformation was conducted on continuous variables to standardize the values into z scores, which facilitated interpretation and allowed us to compare covariates.

#### Individual-level covariates

Covariates at the individual level that could potentially act as confounders include sex, age, total household income (less than $15,000, $15,000 to less than $25,000, $25,000 to less than $35,000, $35,000 to less than $50,000, and equal to or greater than $50,000), race/ethnicity (White, African American, Asian, Hispanic and Other), education (less than high school, high school, post-secondary, and graduate school), and marital status (couple or single). The metropolitan statistical area was used to determine the type of geographical setting in which a subject resided in. The setting was categorized into urban (within the central city of the metropolitan statistical area), suburban (within the metropolitan statistical area, but not within the central city) and rural (not in the metropolitan statistical area).

*Gender*. The BRFSS measured the biological sex (females and males). However, given that sex and gender, i.e. the socially constructed "femaleness" or "maleness" in a society are inextricably interconnected and reciprocally influence each other [31] and that sex is being investigated in relation with different behaviors that are known to vary greatly and influenced by gender [32], for this investigation, we interpreted and discussed findings while integrating gender.

### Outcome measures

Fruit and vegetable consumption was assessed from the six-item fruit and vegetable frequency questionnaire, which is part of the Behavioral Risk Factor Surveillance System rotating core survey questionnaire, administered every other year. The interviewer asked the respondents how many times per day, week, or month they consumed 100% fruit juice, whole fruits, dried beans, dark green vegetables, orange vegetables, and other vegetables over the previous month. Daily consumption was computed from summed responses divided by seven for weekly frequencies, 30 for monthly frequencies, and 365 for yearly frequencies. Fruit and vegetable consumption was computed from responses to all 6 questions, total daily frequency of fruit consumption comprised of responses about intake of fruit juice and fruit consumption, total daily vegetable consumption was obtained from the responses to questions on dried beans, dark green vegetables, orange vegetables and other vegetables, and lastly, fruit juice intake was simply calculated from the frequency of 100% fruit juice consumption. These four outcome measures were dichotomized into (1 = yes or 0 = no) whether consuming fruits or vegetables five times daily, fruits twice a day, vegetables three times a day, or fruit juice once a day, based on the U.S. Department of Agriculture dietary intake recommendation (https://www.usda.gov/topics/food-and-nutrition/dietary-health).

### Statistical analyses

Multilevel logistic regression was used to investigate the association between state-level income inequality and the likelihood of meeting fruit and vegetable recommendations. The following sequences of analyses were performed in order to fit the appropriate models [33]. The first set of analyses involved estimating a state-level intercept-only model, and the 95% plausible value range, which represents the degree of variability between US states for each dietary outcome. Second, the crude relationship between income inequality and each dietary outcome was then estimated. The third stage of analysis involved adding both individual-level and state-level demographic characteristics into the models (model 1). Finally, a cross-level gender by state-level income inequality interaction term was included in the model (model 2) [33]. This interaction term help to determine if the association of income inequality differed by gender. The cross-level interactions of state income inequality and household income and state income inequality and race were not significant, which indicates that state inequality did not have a differential association on level of income nor on racial background (results not reported).

The Behavioral Risk Factor Surveillance System sampling weights were used, and analyses were conducted with SAS version 9.4 (SAS Institute Inc., Carey, NC) and HLM 6.04 (Hierarchical Linear Modeling, Scientific Software International, Chicago, IL, USA).

## Results

The 2013 Behavioral Risk Factor Surveillance System dataset included 483,865 respondents from 50 states and the District of Columbia. All respondents with missing data on six-item fruit and vegetable frequency questionnaire and other covariates were excluded, yielding a case-complete dataset of 270,612 participants (55.9%). Those excluded were less likely to be white, female, older, and from urban settings.

The characteristics of the respondents with complete data are described in Table 1. Among the respondents, 39.7% were men. Most of the participants were white (78.8%), followed by Hispanic (7.9%), African American (8.4%), Asian (2.2%), Other (1.9%) and Native (0.8%). Of the respondents, 34.0%, 25.9%, 20.3%, 11.8%, 5.5%, and 2.5% were aged ≥65 years, 55–65 years, 45–54 years, 35–44 years, 25–34 years and 18–24 years, respectively. Household income level was distributed as 9.4% in ‘less than $15,000’, 15.5% in ‘$15,000 to $25,000’, 10.4% in ‘$25,000 to $35,000’, 14.1% in ‘$35,000 to $50,000’ and 50.6% in ‘greater than $50,000’, respectively. Over half of the population lived in urban settings (62.8%).

**Table 1:** Characteristics of US adults participating in the 2011 Behavioral Risk Factor Surveillance System (BRFSS) (n = 270,612) and US states (50 states and the District of Columbia).

	Unweighted n	Weighted		
Individual Level Characteristics		percentage		
Sex				
Male	104,849	39.7		
Female	165,763	60.3		
Age, years				
18–24	4,358	2.5		
25–34	14,520	5.5		
35–44	29,716	11.8		
45–54	48,481	20.3		
55–64	70,275	25.9		
≥65	103,262	34.0		
Racial Background				
White	223,778	78.8		
African American	21,083	8.4		
Native	4,092	0.8		
Asian	3,901	2.2		
Hispanic	12,017	7.9		
Other	5,741	1.9		
Household Income				
Less than 15,000	28,820	9.4		
15,000 to 25,000	46,681	15.5		
25,000 to 35,000	31,149	10.4		
35,000 to 50,000	40,390	14.1		
Greater than 50,000	123,572	50.6		
Education				
Less than High School	18,821	7.7		
High School	75,958	26.2		
Some College	73,392	26.2		
College	102,441	39.9		
Marital Status				
Couple	156,144	67.1		
Single	114,468	32.9		
Setting				
Urban	132,808	62.8		
Suburban	41,025	17.3		
Rural	96,779	19.9		
State Level Characteristics (n = 51)	Mean (SD)	Median	Interquartile Range	Range
Gini Coefficient	0.45 (0.02)	0.45	0.028	0.419–0.529
State Median Income, USD	51,189 (8,524)	49,687	12210	37,838–70,976
Proportion of Black	12.1 (11.1)	8.7	13.3	0.8–52.2
Proportion of Poor	14.8 (3.2)	14.6	4.8	8.3–22.4
State Population	6,053,834 (6,823,984)	4,339,367	5,156,958	563,626–37,253,956

The characteristics of the 50 States and District of Columbia are presented in Table 1. The mean, standard deviation, median, Interquartile Range, and range of the Gini coefficient are 0.45 (SD = 0.02), 0.45, 0.028, and 0.42–0.53, respectively. The state median income, proportion black, proportion poor, state population are $51,189 (SD = $8,524), 12.1% (SD = 11.1%), 14.8% (SD = 3.2%), and 6,053,834 (SD = 6,823,984), respectively.

The intercept-only multi-level models confirmed that there was significant variability on our outcomes of interest across US states. The overall predictive probability and the plausible value ranges are summarized in Table 2. For example, the intercept-only model indicated that the probabilities of adults consuming recommended amounts of dietary outcomes across the US states are as follows; 5%-15% for ≥5 times of fruits and vegetables daily, 21%-41% for ≥2 times of fruits daily, 10%-22% for ≥3 times of vegetables daily, and 72%-95% for fruit juice daily.

**Table 2:** The overall predicted probability of meeting fruits and vegetables dietary intake guidelines and fruit juice consumption across US states and the plausible value range, which describes the range within each the predicted probability varies across US states.

	Overall Predictive Probability (%)	Plausible Value Range
Outcome		
Fruits and Vegetables ≥5 Daily	0.09	0.05–0.15
Fruits ≥2 Daily	0.31	0.21–0.41
Vegetables ≥3 Daily	0.16	0.10–0.22
Fruit Juice Daily	0.78	0.72–0.95

**Fig 1:**
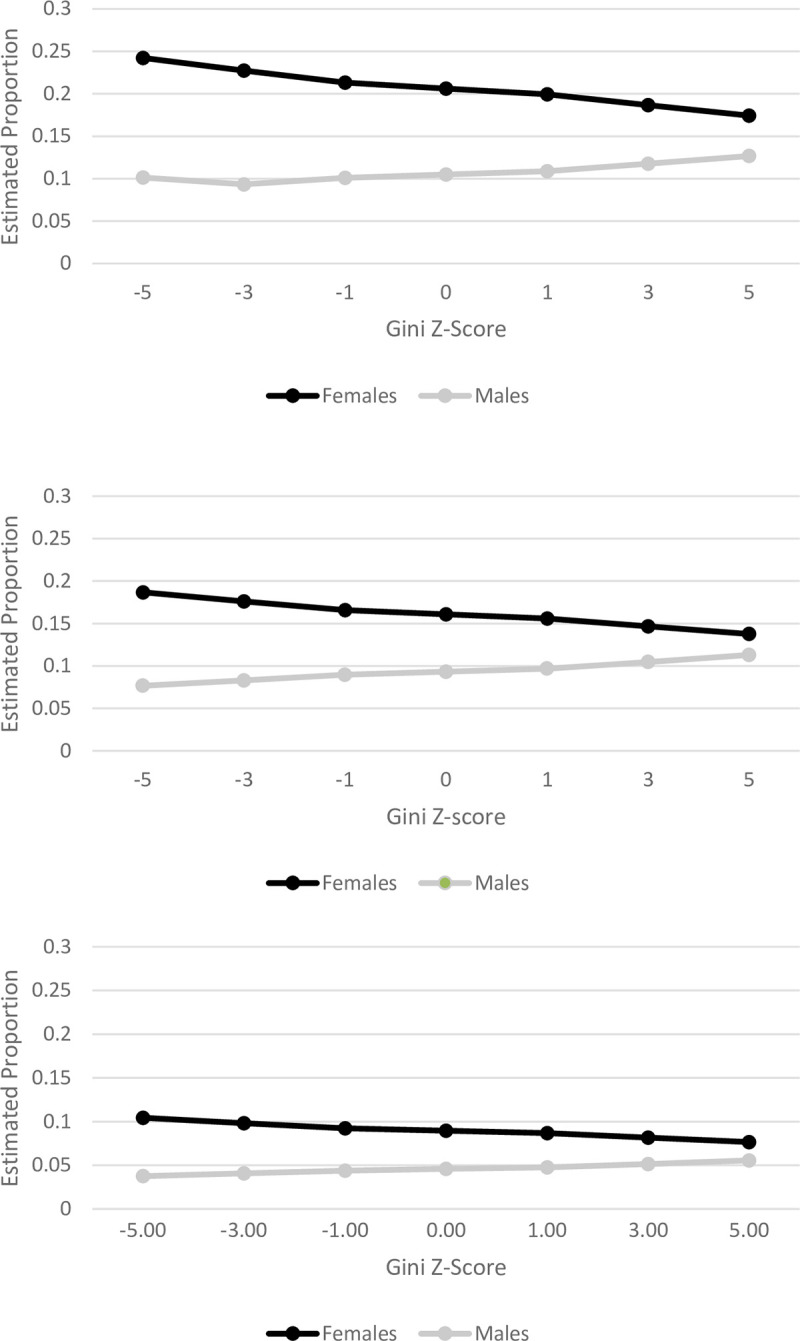
Estimated proportion of women and men who consume a) 2 fruits a day, b) 3 vegetables a day, and c) Five Fruits and vegetables per day.

The results of analyses for the associations between income inequality and outcomes on fruit and vegetable consumption are presented in Table 3. Model 1 includes the individual-level covariates and state-level covariates. No significant relationship between income inequality and fruit and vegetable consumption was observed. For daily fruit juice intake, the model indicated for every increase in standard deviation of state Gini coefficient (OR = 1.07; 95% CI: 1.03–1.11), there was an increased likelihood for daily fruit juice consumption. In model 2, the cross-level interaction of state income inequality and sex was tested. The results from model 2 indicated that, among women, a standard deviation in Gini coefficient was associated with a decreased likelihood to consume ≥5 times of fruits and vegetables daily (OR = 0.93; 0.87–0.99), ≥2 times of fruits daily (OR = 0.95; 95% CI, 0.92–0.99), ≥3 times of vegetables daily (OR = 0.92; 95% CI, 0.89–0.96). The significant gender-income inequality cross-level interaction is displayed in Fig 1.

**Table 3:** Cross-sectional adjusted associations between income inequality and odds for meeting fruit and vegetables, fruit, vegetable recommendations, and daily fruit juice consumption among participants in the 2013 Behavioral Risk Factor Surveillance System (BRFSS).

	Fruits and vegetables ≥5 times daily				Fruits ≥2 times daily				Vegetables ≥3 times daily				Fruit juice daily			
	Adjusted		Adjusted + Interaction		Adjusted		Adjusted + Interaction		Adjusted		Adjusted + Interaction		Adjusted		Adjusted + Interaction	
	OR	95%CI	OR	95%CI	OR	95%CI	OR	95%CI	OR	95%CI	OR	95%CI	OR	95%CI	OR	95%CI
Intercept	0.05	(0.04,0.06)	0.05	(0.04,0.06)	0.12	(0.09,0.16)	0.12	(0.09,0.16)	0.11	(0.08,0.13)	0.10	(0.08,0.13)	0.14	(0.12,0.18)	0.14	(0.11,0.18)
**State Characteristics**																
Gini (Z-Score)	0.99	(0.92,1.07)	1.04	(0.96,1.14)	0.97	(0.86,1.10)	1.01	(0.89,1.14)	0.99	(0.94,1.05)	1.04	(0.98,1.11)	1.07	(1.03,1.11)	1.08	(1.02,1.14)
State Median Income (Z-Score)	1.02	(0.91,1.14)	1.02	(0.91,1.14)	1.05	(0.87,1.28)	1.05	(0.87,1.28)	1.03	(0.96,1.10)	1.03	(0.96,1.10)	0.95	(0.90,1.00)	0.95	(0.90,1.00)
Population Size (Z-Score)	1.1	(1.04,1.16)	1.1	(1.04,1.16)	1.09	(1.01,1.17)	1.09	(1.01,1.17)	1.07	(1.03,1.11)	1.07	(1.03,1.11)	1.03	(1.01,1.04)	1.03	(1.01,1.04)
Proportion Black (Z-Score)	1.05	(0.96,1.15)	1.05	(0.96,1.15)	1.01	(0.85,1.21)	1.01	(0.85,1.21)	1.03	(0.98,1.09)	1.03	(0.98,1.09)	1.04	(1.01,1.07)	1.04	(1.01,1.07)
Proportion in Poverty (Z-Score)	0.96	(0.83,1.11)	0.96	(0.82,1.11)	0.98	(0.77,1.26)	0.98	(0.77,1.26)	1.01	(0.92,1.10)	1.01	(0.92,1.10)	0.89	(0.83,0.95)	0.89	(0.83,0.95)
Census Division (ref: New England)	1.00		1.00		1.00		1.00		1.00		1.00		1.00		1.00	
Middle Atlantic	0.74	(0.63,0.88)	0.74	(0.63,0.89)	0.81	(0.66,1.00)	0.81	(0.66,1.00)	0.81	(0.71,0.92)	0.81	(0.71,0.92)	0.86	(0.80,0.92)	0.86	(0.80,0.92)
East North Central	0.79	(0.64,0.98)	0.79	(0.64,0.98)	0.96	(0.73,1.28)	0.97	(0.73,1.28)	0.82	(0.69,0.96)	0.82	(0.69,0.97)	0.78	(0.72,0.85)	0.78	(0.72,0.85)
West North Central	0.77	(0.63,0.94)	0.77	(0.63,0.94)	0.9	(0.71,1.14)	0.9	(0.71,1.14)	0.78	(0.66,0.91)	0.78	(0.66,0.91)	0.78	(0.70,0.86)	0.78	(0.70,0.86)
South Atlantic	0.7	(0.53,0.93)	0.7	(0.53,0.93)	0.67	(0.45,1.00)	0.67	(0.45,1.00)	0.83	(0.69,1.00)	0.83	(0.69,1.00)	0.77	(0.71,0.85)	0.77	(0.71,0.85)
East Coast Central	0.5	(0.36,0.71)	0.5	(0.36,0.71)	0.40	(0.22,0.73)	0.40	(0.22,0.73)	0.7	(0.54,0.90)	0.7	(0.55,0.90)	0.74	(0.67,0.82)	0.74	(0.67,0.82)
West South Central	0.55	(0.43,0.72)	0.55	(0.43,0.72)	0.47	(0.33,0.68)	0.47	(0.33,0.68)	0.69	(0.56,0.85)	0.69	(0.56,0.85)	0.66	(0.59,0.74)	0.66	(0.59,0.74)
Mountain	0.99	(0.81,1.23)	1.00	(0.81,1.23)	0.99	(0.77,1.29)	0.99	(0.77,1.29)	1.00	(0.85,1.17)	1.00	(0.85,1.17)	0.79	(0.71,0.88)	0.79	(0.71,0.88)
Pacific	1.02	(0.78,1.33)	1.02	(0.79,1.34)	1.07	(0.77,1.48)	1.07	(0.77,1.48)	1.12	(0.90,1.39)	1.12	(0.90,1.39)	0.70	(0.64,0.76)	0.70	(0.64,0.76)
**Individual Characteristics**																
Sex (ref: male)	1.00		1.00		1.00		1.00		1.00		1.00		1.00		1.00	
Female	1.98	(1.86,2.11)	2.05	(1.92,2.19)	2.17	(2.09,2.26)	2.22	(2.13,2.31)	1.8	(1.71,1.89)	1.86	(1.77,1.96)	0.79	(0.76,0.83)	0.80	(0.77,0.82)
Gini Z-Score			0.93	(0.87,0.99)			0.95	(0.92,0.99)			0.92	(0.89,0.96)			0.99	(0.94,1.04)
Age (years)	1.01	(1.00,1.02)	1.01	(1.00,1.02)	1.02	(1.01,1.03)	1.02	(1.01,1.03)	0.99	(0.98,1.00)	0.99	(0.98,1.00)	1.23	(1.20,1.26)	1.23	(1.20,1.26)
Household Income (ref: less than 15,000)	1.00		1.00		1.00		1.00		1.00		1.00		1.00		1.00	
15,000 to 25,000	1.03	(0.93,1.14)	1.03	(0.93,1.14)	1.06	(0.99,1.13)	1.06	(0.99,1.13)	1.01	(0.94,1.09)	1.01	(0.94,1.09)	1.02	(0.96,1.08)	1.02	(0.96,1.08)
25,000 to 35,000	1.04	(0.96,1.13)	1.04	(0.96,1.13)	1.1	(1.02,1.18)	1.1	(1.02,1.18)	1.05	(0.94,1.18)	1.05	(0.94,1.18)	0.98	(0.90,1.07)	0.98	(0.90,1.07)
35,000 to 50,000	1.08	(0.98,1.18)	1.08	(0.98,1.18)	1.2	(1.13,1.28)	1.2	(1.13,1.28)	1.09	(1.00,1.17)	1.09	(1.00,1.17)	0.93	(0.87,1.00)	0.93	(0.87,1.00)
Greater than 50,000	1.22	(1.12,1.34)	1.22	(1.12,1.34)	1.37	(1.26,1.48)	1.37	(1.26,1.48)	1.23	(1.11,1.35)	1.23	(1.11,1.35)	0.80	(0.73,0.88)	0.80	(0.73,0.88)
Education (ref: no high school)	1.00		1.00		1.00		1.00		1.00		1.00		1.00		1.00	
High school	0.93	(0.82,1.06)	0.93	(0.82,1.06)	0.93	(0.82,1.05)	0.93	(0.82,1.05)	0.90	(0.82,0.98)	0.90	(0.82,0.98)	0.98	(0.94,1.03)	0.98	(0.94,1.03)
Attended college	1.29	(1.09,1.52)	1.28	(1.09,1.51)	1.13	(0.97,1.32)	1.13	(0.97,1.32)	1.23	(1.10,1.39)	1.23	(1.10,1.38)	1.03	(0.97,1.09)	1.03	(0.97,1.09)
College Graduate	1.82	(1.59,2.08)	1.82	(1.59,2.08)	1.59	(1.40,1.80)	1.59	(1.40,1.80)	1.62	(1.46,1.81)	1.62	(1.46,1.81)	1.19	(1.12,1.27)	1.19	(1.12,1.27)
Race (ref: white)	1.00		1.00		1.00		1.00		1.00		1.00		1.00		1.00	
African American	1.01	(0.94,1.10)	1.01	(0.94,1.10)	1.00	(0.91,1.08)	1.00	(0.91,1.09)	0.87	(0.81,0.93)	0.87	(0.82,0.93)	1.47	(1.39,1.55)	1.47	(1.39,1.55)
Asian	1.04	(0.87,1.26)	1.04	(0.87,1.25)	0.93	(0.80,1.09)	0.93	(0.80,1.08)	1.2	(1.03,1.40)	1.2	(1.03,1.39)	0.92	(0.82,1.04)	0.92	(0.82,1.04)
Native	1.26	(1.01,1.58)	1.27	(1.02,1.58)	0.95	(0.80,1.14)	0.95	(0.80,1.14)	1.11	(0.94,1.33)	1.11	(0.94,1.33)	1,28	(1.11,1.48)	1.28	(1.11,1.48)
Hispanic	1.46	(1.32,1.61)	1.46	(1.32,1.61)	1.24	(1.11,1.39)	1.24	(1.11,1.39)	1.35	(1.22,1.49)	1.35	(1.22,1.49)	1.33	(1.22,1.44)	1.33	(1.22,1.44)
Other	1.45	(1.28,1.65)	1.46	(1.28,1.65)	1.15	(1.05,1.27)	1.16	(1.05,1.27)	1.41	(1.25,1.60)	1.42	(1.25,1.61)	1.34	(1.21,1.49)	1.34	(1.22,1.49)
Marital status (ref: coupled)	1.00		1.00		1.00		1.00		1.00		1.00		1.00		1.00	
Single	0.90	(0.87,0.93)	0.90	(0.87,0.93)	0.89	(0.86,0.92)	0.89	(0.86,0.92)	0.91	(0.88,0.94)	0.91	(0.88,0.94)	1.10	(1.05,1.14)	1.10	(1.05,1.14)
Setting (ref: Rural)	1.00		1.00		1.00		1.00		1.00		1.00		1.00		1.00	
Urban	1.08	(1.02,1.15)	1.08	(1.02,1.15)	1.12	(1.07,1.18)	1.12	(1.07,1.18)	1.04	(1.00,1.08)	1.04	(1.00,1.08)	1.03	(0.99,1.07)	1.03	(0.99,1.07)
Suburban	1.03	(0.96,1.09)	1.03	(0.97,1.09)	1.07	(1.01,1.13)	1.07	(1.01,1.13)	0.98	(0.95,1.02)	0.98	(0.95,1.02)	1.04	(0.99,1.10)	1.04	(0.99,1.10)

## Discussion

To our knowledge, this is the first study to show the gendered association between state-level income inequality and dietary behavior, particularly fruit and vegetable consumption. Using data from a nationally representative survey of the US adults, we observed that high income inequality was significantly associated with decreased odds of meeting fruit and vegetable recommendations among women. Also, as the level of income inequality increased, increased odds of drinking fruit juice were observed among the US adults. In addition, our study shows that there is no evidence of a significant cross-level interaction between income inequality and individual-level income; thus, the association between inequality and the odds of meeting fruit and vegetable recommendation or frequency of fruit juice did not differ across incomes among women.

Previous studies examining the relationship between income inequality and dietary behavior are sparse. However, the current study is consistent with studies suggesting income inequality is associated with other health behaviors such as physical activity [19] and dietary-related chronic conditions and diseases [19, 20, 23]. An ecological study conducted with 21 developed countries showed that income inequality was significantly related to obesity both among men and women, diabetes mortality and average calorie consumption per capita per day [21]. Studies utilizing individual-level data conducted within the U.S. have shown significant associations between state level income inequality and abdominal weight gain in men, increased odds of sedentary lifestyles among both men and women, and higher BMI among women [23, 34], and odds for not meeting physical activity recommendations among women [19]. In comparison to fresh fruit, fruit juice is more affordable, has a longer shelf life, and more likely to be available within corner stores. This might explain our observation that income inequality was associated with an increased likelihood for consuming fruit juice.

Our findings regarding the cross-level interaction of state-level income inequality and gender are noteworthy. Several studies in the past suggest that the effects of macro-level factors on health outcome may differ by gender [19, 23, 35, 36]. For example, empirical evidence indicates that the relationship between income inequality and obesity is stronger for women than for men [24]. The potential consequences and benefits of policies that drive income inequality might influence the dietary behavior of women more profoundly than it does for men. The inverse association between state-level income inequality and fruit juice consumption found in this study should also be highlighted. Previous literature found higher consumption of 100% fruit juice among lower income households and racial minorities [37], potentially driven by price, access, and insufficient reach of nutrition education efforts to warn consumers that fruit juice is not the best substitute for soda.

Two potential pathways may explain the association of income inequality and fruit and vegetable consumption among women. The first mechanism comes from a political aspect in which a society with greater income inequality tends to invest less on human capital and allocates less public funding for social goods such as education, health services, social welfare and food assistant programs [16, 38]. This may lead to adverse education, nutrition, and overall health outcomes, affecting disproportionately vulnerable populations such as women [16, 38]. In the US, social welfare policies are under the jurisdiction of the state. For example, welfare and SNAP benefits are distributed by each US state with variations in the administrative costs and participation rates across the US states [39]. If welfare and nutritional support is diminished, individuals and families from lower socioceconomic backgrounds may have less money and resources for nutritious and quality food. When individuals and families have little money and resources, they are more likely to afford food that is caloric dense food and unhealthy, over nutritious food such as fresh fruit and vegetables [40].

Furthermore, researchers have observed that U.S. states with greater income inequality had higher rates of unemployment, and had higher proportions of their population receiving income assistance and food stamps [41]. The U.S. had one of the highest poverty rates among developed countries, affecting more women than men and leading to greater prevalence of food insecurity among female-headed households [42, 43]. Households with food insecurity are reported to consume less fruits and vegetables than food secure households [10, 44]. It is possible that women living in the society with greater income inequality experience lower consumption of fruits and vegetables, due to higher prevalence of food insecurity in that population. The Supplemental Nutrition Assistance Program (SNAP), formerly known as food stamps, are distributed by each U.S. state that provides food-purchasing assistance for low and no-income people [39]. Although research indicates that SNAP participants consume approximately the same amount of calories in comparison to higher income individuals and families, the food they consume is not necessarily healthy, choosing to eat foods high in starch, fat, and sugar, over fresh fruits and vegetables [40]. Researchers further explain this is due to SNAP participants having less money, and as a result eat fewer meals, which are less healthy, in order to compensate [40]. However, this cannot be the only explanation for the association between income inequality and fruit and vegetable consumption among women, since such association appears to be persistent even after carefully controlling for individual socioeconomic status.

Second, the association between income inequality and fruit and vegetable consumption among women can be explained by “psychosocial theory” of income inequality and health [24]. Income inequality has been hypothesized to lead to adverse mental health outcomes, such as psychosocial stress, which results from the shame, loss of self-respect and invidious social comparisons and through the erosion of social cohesion [22, 38, 45]. Wilkinson and Pickett argued that when the income gap between the rich and poor widens, it heightens feelings of insecurity and shame among members of society who are left behind [24]. For example, low social status could increase anxiety and stress levels and reduce people’s ability to exercise self-control over their lifestyle choices [21]. In response to psychosocial stress, men and women are known to perceive, react and cope with their social and physical environments differently [35]. Several studies have shown that women are at greater risk for depression in income inequality states and are more susceptible to stress-related disordered eating than men [36, 46, 47]. As a result of psychosocial stress stemming from living in areas with high income inequality, as part of a coping mechanism, women’s dietary behavior may be affected, resulting in less consumption of fruits and vegetables [21, 48, 49]. Combined, the results of the study warrant future investigation of the association between income inequality and fruit and vegetable consumptions stratified by gender. Such investigation should also incorporate analyzing the complexity of intersectionality that connect political, social, and psychosocial factors to explain a myriads of adverse health outcomes that impact individuals differently based on their social positions.

The results of this study should be interpreted with caution in light of the following limitations. First, measurement of the outcome was based on self-report, which is prone to misclassification due to recall bias. However, the extent that such misclassification should be unrelated to our exposure outcome; thus, the resulting bias is likely to have attenuated our results. Secondly, the difference between the Behavioral Risk Factor Surveillance System module and other methods of dietary assessment should be considered. The Behavioral Risk Factor Surveillance System module assesses frequency of intake (times per day) rather than servings; thus it is insensitive to changes in serving size [50]. However, previous research has shown good agreement between the Behavioral Risk Factor Surveillance System estimates and other methods of dietary intake, such as multiple diet records or recalls [51]. Third, in general, estimates of fruit and vegetable consumption from abbreviated food frequency questionnaires, such as the Behavioral Risk Factor Surveillance System module, are lower than those from other methods of dietary assessment [50]. Estimates of fruit and vegetable consumption based on six-item food frequency questionnaires, used in the Behavioral Risk Factor Surveillance System, are generally lower when compared to interviewer administered 24-hour recall, which provides better quality and a less biased data for a single day [52]. Fourth, fried potatoes, such as French fries, are excluded from the Behavioral Risk Factor Surveillance System module. The exclusion of fried potatoes could also contribute to lowering the estimates of overall intake compared with the modules that included these food items. Finally, since a large proportion of the sample were removed due to missing data, a selection bias could have been introduced. Therefore, estimates may be biased because our analytical sample disproportionately excluded males and individuals from lower household income backgrounds. This potential selection bias may explain insignificant associations between income inequality and fruit and vegetable intake among men.

In summary, this study demonstrates that state-level income inequality is a correlate of fruit and vegetable consumption among women. In more unequal states, women were significantly less likely to meet fruit and vegetable recommendations, and both men and women were more likely to drink fruit juice compared to their counterparts in more equal states. Research is needed to further examine the longitudinal relationship between income inequality and dietary intake, which will allow public health researchers to determine if this relationship is causal. Also, future research should explore the underlying pathways in order to gain a better understanding of the relationship between state-level income inequality and dietary behavior while paying attention to the moderating role of gender. For instance, further research is needed to understand the relationships of income inequality with state variations in food insecurity, food assistance policies and dietary behavior. Lastly, it is conceivable that consumption of fruits and vegetables not only functions as a dietary marker, but also infers the well-being of the population in terms of socioeconomic status, human capital and social prosperity. Taken together, this current study suggests that diet may be one of the mechanisms through which income inequality affects chronic conditions, particularly among women.

## List of abbreviations

BMI: Body Mass Index
BRFSS: Behavioral Risk Factor Surveillance System
OR: Odds Ratio
SNAP: The Supplemental Nutrition Assistance Program
